# Lumpy Skin Disease Virus Infected Calves Showing Multisystemic Vasculitis on Postmortem Examination: A Summary of Six Cases

**DOI:** 10.1155/tbed/8721034

**Published:** 2026-01-06

**Authors:** Israt Jerin, Md. Riabbel Hossain, Shadia Tasnim, Seikh Masudur Rahman, Anja Globig, Bernd Hoffmann, Emdadul Haque Chowdhury, Rokshana Parvin

**Affiliations:** ^1^ Department of Pathology, Faculty of Veterinary Science, Bangladesh Agricultural University, Mymensingh, Bangladesh, bau.edu.bd; ^2^ Institute of International Animal Health/One Health, Friedrich-Loeffler-Institute, Greifswald-Insel Riems, Germany, fli.de; ^3^ Institute of Diagnostic Virology, Friedrich-Loeffler-Institute, Greifswald-Insel Riems, Germany, fli.de

**Keywords:** calf mortality, complication, genomic divergence, LSD, multi-systemic spread, pathology, vasculitis

## Abstract

Lumpy skin disease (LSD) is a rapidly spreading transboundary viral disease of cattle and water buffalo that poses a significant threat to livestock health and economies of Bangladesh. Calf mortality is steadily increasing over time. This study documented fatal calf mortality with vasculitis‐driven multisystemic pathology, which has been rarely reported in Bangladesh. To investigate the rising incidence of calf mortality in Bangladesh, this study conducted a pathological investigation of six deceased calves and molecular analyses of the viruses. Clinically affected calves in north‐central Bangladesh exhibited high fever, skin nodules, lymphadenopathy, joint swelling, respiratory distress, ocular and nasal discharge, and edema. Cutaneous nodules often sloughed off, leaving deep ulcerative lesions. Gross pathology of six deceased calves revealed multisystemic lesions, including congestion and edema of the nasal passages, tracheitis, pulmonary consolidation, renal congestion and necrosis, hepatomegaly with multifocal necrosis, splenic atrophy, and lymphadenopathy. Histopathology demonstrated necrotizing inflammation, severe broncho‐interstitial pneumonia, hepatic centrilobular necrosis, myocardial infarction, interstitial nephritis with vasculitis, and marked lymphoid depletion. Molecular detection confirmed moderate to high viral loads in the skin and internal organs, consistent with the pathological findings. Whole‐genome phylogenetic analysis placed the isolates within cluster 1.2 (classical African/Kenyan sheep and goat pox [KSGP]‐like lineage), with one strain clustering closely with isolates from India, Serbia, and Russia, indicating possible cross‐border viral movement and genetic evolution. These findings confirm the continued circulation of classical cluster 1.2 LSD virus (LSDV) in Bangladesh, with accumulating genetic variation possibly enhancing virulence in calves. The study underscores the need for sustained genomic surveillance, expanded vaccination, and improved biosecurity to mitigate future LSD outbreaks.

## 1. Introduction

Lumpy skin disease (LSD) is a highly contagious and economically significant transboundary viral disease of cattle caused by infection with the LSD virus (LSDV), a member of the Capripoxvirus genus within the family Poxviridae [1–3]. It was initially geographically confined to sub‐Saharan Africa but spread rapidly to the Middle East, Central Asia, and, more recently, to South and Southeast Asia, including Bangladesh [3, 4]. The disease is characterized by fever, nodular dermatitis, lymphadenopathy, lameness, reduced milk yield, reproductive failure [5, 6], edematous swelling of the limbs, and, in some cases, severe respiratory distress and lameness [2, 7]. In severe cases, the disease can be fatal. Although traditionally regarded as a dermatotropic infection, emerging evidence indicates that LSDV induces a broader range of systemic pathological changes, including severe internal organ necrosis [8]. The first outbreak of LSD in Bangladesh was reported in 2019 [9] and the disease became endemic since then, leading to significant morbidity and mortality in cattle populations [10, 11]. Recently, severe cases of LSD presentations have increasingly been reported, particularly in calves and immunocompromised animals [12]. These manifestations include respiratory distress, diarrhea, hepatic failure, and sudden death, suggesting involvement of internal organs other than cutaneous lesions [2, 13]. Histopathological findings are most frequently observed in the skin, with cutaneous nodules being a common manifestation [14]. These lesions typically exhibit features of dermatitis characterized by mononuclear cell infiltration, the presence of intracytoplasmic inclusion bodies, disruption of connective tissue in the reticular dermis, vasculitis, dermal necrosis accompanied by hemorrhage, and vascular congestion [2]. However, histopathological evidence of lesions in internal visceral organs remains scarce and underreported. The reason behind the systemic pathogenesis of LSDV would likely be a consequence of its marked epithelial celltropism [15]. The virus also appears to suppress host immunity, rendering infected animals vulnerable to secondary infections that exacerbate disease outcomes [16]. This immune suppression creates a favorable environment for the virus to evolve and adapt [17]. Simultaneously, the emergence of more virulent LSDV strains across Asia has heightened interest in the virus’s genomic evolution. Recent past studies have reported characteristic mutations, insertions, deletions, and recombination events between field and vaccine strains circulating in the region [18]. Such genomic divergence may influence viral virulence, tissue tropism, transmission dynamics, and host susceptibility [18], factors potentially contributing to the increased disease severity and mortality observed in Bangladesh. The widespread use of heterologous or partially attenuated vaccines under field conditions may be exerting selective pressure on LSDV, facilitating the emergence of more pathogenic variants [19]. In Bangladesh, a recent study demonstrated short‐term seroconversion in both vaccinated animals and those that recovered from natural infection [20]. In this context, a deeper understanding of the evolving pathogenic profile and immune evasion mechanisms of the virus is essential. Comprehensive characterization of the genomic features of circulating LSDV strains and their association with systemic complications is critical for effective disease control and vaccine development. Elucidating the underlying causes of calf mortality and the exacerbation of systemic disease is crucial to guide targeted intervention strategies. Therefore, the present study aimed to bridge this knowledge gap by conducting a detailed pathological and molecular investigation of LSDV infections in Bangladesh. This included an assessment of clinical signs, gross and histopathological changes, particularly necrosis and inflammation in vital internal organs, as well as whole‐genome sequencing coupled with phylogenetic analysis of field LSDV strains.

## 2. Materials and Methods

### 2.1. Case Investigation

The study was conducted from December 2023 to May 2025 in the Trishal, Bhaluka, Tarakanda, and Sadar upazilas (subdistricts) of the Mymensingh district, northern Bangladesh, where multiple LSD outbreaks were reported by farmers and government veterinarians. The study included calves from households as well as small‐to large‐scale farms. In this study, farms were categorized based on herd size as follows: households with fewer than five calves, small‐scale farms with 5–50 calves, and large‐scale farms with more than 50 calves. Typically, cattle from household and small‐scale farms were unvaccinated. While the outbreak occurred in a large‐scale herd, the adult animals had been vaccinated against LSDV; however, the affected calves were unvaccinated at the time of infection. Whenever outbreaks were identified, clinical investigations were carried out.

### 2.2. Necropsy, Gross Examination, and Sample Collection

The study examined six calves that died during LSD outbreaks (Table 1), reported by farmers or veterinarians. The six calves included in this study were not randomly selected but were all available fatal cases submitted to the Department of Pathology, Bangladesh Agricultural University, during the 2024 LSD outbreak. These cases were chosen based on the presence of typical clinical and gross pathological lesions consistent with LSD, confirmed by PCR. Postmortem examinations were conducted in the field and at the laboratory of the Department of Pathology, including external examinations of skin lesions and systemic, systematic dissection for gross evaluation and sample collection for molecular and histopathological analyses. Organs sampled included skin nodules, nasal passages, trachea, lungs, heart, rumen, abomasum, intestines, liver, kidneys, spleen, and lymph nodes. Samples for molecular analyses were transported to the laboratory under cold chain and stored at −80°C until testing.

**Table 1:** Clinical characteristics of six calves affected by LSD.

Case no.	Age	Breed	Source	Location	Disease onset	Major clinical signs	Treatment given	Died on
Case‐1	1.5 months	Holstein Friesian cross	Household	Akua, Mymensingh	December 27, 2023	Multiple nodules (mostly 1–3 cm in diameter) in the whole body, swollen hock, stifle, and fetlock joint, severe respiratory distress due to blockade of the nasal passage and conjunctivitis	Ceftiofur sodium‐6 days, pheniramine maleate‐6 days, and meloxicam‐6 days	January 03, 2024
Case‐2	3 months	Holstein Friesian cross	Household	Kathal, Trishal, Mymensingh	December 31, 2023	Multiple nodules in the mandibular region (mostly 1–2 cm in diameter), severe respiratory distress due to blockade of the nasal passage and conjunctivitis	Amoxicillin‐6 days, pheniramine maleate‐6 days, and flunixin meglumine‐6 days	January 06, 2024
Case‐3	2 months	Sahiwal cross	Small‐scale farm	Bhaluka, Mymensingh	September 26, 2023	Multiple nodules in the whole body (mostly 1–4 cm in diameter), severe respiratory distress, conjunctivitis, and swelling of joints	Amoxicillin‐7 days, pheniramine maleate‐7 days, and flunixin meglumine‐7 days	October 04, 2023
Case‐4	2.5 months	Holstein Friesian cross	Large‐scale farm	Tarakanda, Mymensingh	September 07, 2024	High fever (105°F), lumps throughout the skin, respiratory distress	Amoxicillin‐6 days, pheniramine maleate‐6 days, flunixin meglumine‐6 days, and vitamin B complex‐6 days as symptomatic treatment	September 14, 2024
Case‐5	1.5 months	Holstein Friesian cross	Large‐scale farm	Trishal, Mymensingh	November 27, 2024	Fever (103°F), the right‐side external part of the mouth is swollen, respiratory distress	Pheniramine maleate‐2 days, flunixin meglumine‐2 days, vitamin B complex‐2, normal Saline‐2 days, and acyclovir‐2 days	November, 2024
Case‐6	3.5 months	Holstein Friesian cross	Large‐scale farm	Trishal, Mymensingh	November 27, 2024	Fever (103°F), different‐sized, multiple raised nodules on the whole body	Pheniramine maleate‐4 days, flunixin meglumine‐4 days, vitamin B complex‐4 days, normal saline‐4 days, zinc‐4 days, and acyclovir‐4 days	December 02, 2024

*Note:* Household: < 5 calves, small‐scale farms: 5–50 calves, and large‐scale farms: > 50 calves. Postmortem examination is done within 3–5 hours of death. The detailed clinical history, including the onset of symptoms, clinical signs, and duration of illness, treatment given, and onset of death, is provided for each case.

### 2.3. Histopathological Examination

Skin nodules, nasal passages, trachea, lungs, heart, rumen, abomasum, intestines, liver, kidneys, spleen, and lymph nodes were processed for histopathology. The samples were fixed in 10% neutral buffered formalin, dehydrated through graded alcohol, cleared with chloroform, and embedded in paraffin. Sections were cut at a thickness of 4 μm and stained with hematoxylin and eosin (H&E) according to a standard procedure. Masson’s trichrome stain was performed to further characterize connective tissue in the rumen. The stained sections were examined under a compound light photomicroscope (Olympus BX43).

### 2.4. DNA Extraction

To detect viral presence, collected tissue samples were placed in 2 mL of appropriate lysis buffer at a 1:10 (w/v) ratio supplemented with streptomycin and penicillin. Each sample was homogenized with a 5 mm stainless steel bead in a 2 mL microcentrifuge tube for 5 min using a TissueLyser II (Qiagen, Hilden, Germany). The homogenate was centrifuged at 8000 × g for 5 min, and 200 μL of the supernatant was transferred to a 1.5 mL microcentrifuge tube for DNA extraction. DNA was extracted using the DNeasy Blood & Tissue Kit (Qiagen, Hilden, Germany) according to the manufacturer’s instructions. The extracted DNA was quantified using a NanoDrop One spectrophotometer (Thermo Scientific, Waltham, MA, USA) and stored at −20°C until further processing.

### 2.5. Molecular Detection

For molecular investigation, the extracted DNA samples were tested using the TaqMan probe‐based qPCR assay that amplified a part of the P32 envelope protein gene using a Luna Universal Probe qPCR Master Mix (NEB, Hitchin, UK). The primers and probe utilized in the molecular detection were Capri‐p32 forward: AAA ACG GTA TAT GGA ATA GAG TTG GGA, Capri‐p32 reverse: AAA TGA AAC CAA TGG ATG GGA TA, and Capri‐p32 FAM: ATG GAT GGC TCA TAG ATT TCC TGA T‐BHQ1 [15, 21]. The qPCR reaction was formulated in a total volume of 12.5 µL, comprising 6 µL of Luna Universal Probe qPCR Master Mix (NEB, Hitchin, UK), 2 µL of primer–probe mix, 2 µL of nuclease‐free water, and 2.5 µL of template DNA. The PCR was conducted on a QuantStudio 5 Real‐Time PCR System (Thermo Fisher Scientific, USA) under the following conditions: one cycle at 95°C for 2 min (activation), followed by 40 cycles at 95°C for 15 s (denaturation) and 60°C for 30 s (annealing and extension) [22]. The cycle threshold (Ct) values obtained at ≤ 35 from the clinical samples were considered positive.

### 2.6. Virus Isolation

qPCR‐positive skin nodules from all six calves were used for virus isolation, and viral loads were quantified by qPCR to confirm the presence of LSDV. Virus inoculum was prepared directly from field‐collected skin nodules, and viral loads were monitored to ensure consistent infection conditions across samples. The inoculum was prepared from the homogenate of skin samples in sterile Dulbecco’s Modified Eagle Medium supplemented with penicillin‐streptomycin and amphotericin B as antibiotics. Confluent monolayers of Madin–Darby bovine kidney (MDBK) cells were utilized for LSDV isolation. A control flask of MDBK cells was always maintained in parallel with the virus‐inoculated flask. The control cells received PBS instead of viral inoculum and were monitored under identical culture conditions. To ensure the validity of the control, these cells were also confirmed to be LSDV‐negative by PCR at the end of the incubation period. The infected cells were monitored daily for 7 days for a cytopathic effect (CPE). The virus was harvested and identified using qPCR as described above.

### 2.7. Sequencing and Phylogeny

LSDV isolates from the skin samples of dead calves were selected for whole‐genome sequencing using Illumina technology, based on prior evidence that skin lesions harbor the highest viral loads. The MasterPure Complete DNA and RNA Purification Kit (Lucigen/Biozym Scientific GmbH, Hessisch Oldendorf, Germany) was used to extract genomic DNA from cell‐culture‐propagated LSDV isolates, following the manufacturer’s instructions. The prepared DNA was submitted to Eurofins Genomics Germany GmbH (Ebersberg, Germany) for high‐throughput sequencing on the Illumina HiSeq 2500 platform (Illumina, San Diego, CA, USA). The sequencing generated over 10 million paired reads for downstream analysis of two LSDV strains.

Consensus sequences were generated using the mapping tool in Geneious v11.1.5 (Biomatters, Auckland, New Zealand), based on several LSDV reference genomes (NC_003027, MH893760, and KX683219). Multiple sequence alignment was performed using the online version of the MAFFT program. Phylogenetic analysis was conducted with the neighbor‐joining method and Jukes–Cantor substitution model within MAFFT, with confidence estimates calculated using 1000 bootstrap replicates [23]. The resulting phylogenetic trees were annotated and visualized using FigTree v1.4.2.

## 3. Results

### 3.1. Clinical Investigations

The most common clinical signs of cattle infected with LSDV included high fever (105°F–106°F), widespread nodular skin swellings, and markedly enlarged lymph nodes. Swelling of the fetlock, hock, and/or carpal joints, causing lameness, was also observed, along with respiratory and nasolacrimal discharge. In two of six dead examined cases, edema appeared in the cranio‐ventral abdominal and thoracic regions, and the tops of skin nodules sloughed off, leaving deep ulcers.

### 3.2. Gross Pathology

Gross pathological examination revealed prominent lesions in multiple organs of all deceased calves. Characteristically, the carcasses exhibited numerous cutaneous nodules distributed across the body surface (Figure 1A). Upon sectioning, individual nodules measured ~2–3 cm in diameter and were firm in consistency (Figure 1B). The prescapular lymph nodes were markedly enlarged, often reaching 4–6 times their normal size (Figure 1C). In addition, one calf (Case 6; Table 1) presented with splenic atrophy characterized by a wrinkled capsule (Figure 1D).

Figure 1Clinical and postmortem findings in calves affected by lumpy skin disease (LSD) virus. The affected animal shows (A) numerous lumps distributed across the entire body surface, (B) deep, well‐demarcated round skin nodules observed upon incision, (C) markedly enlarged prescapular lymph node, and (D) atrophied spleen.

**Figure:**
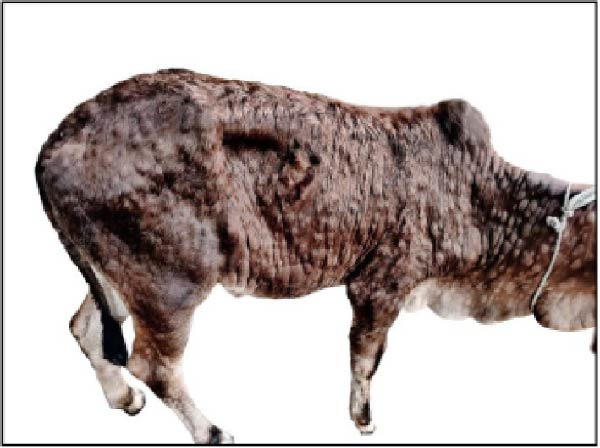
(A)

**Figure:**
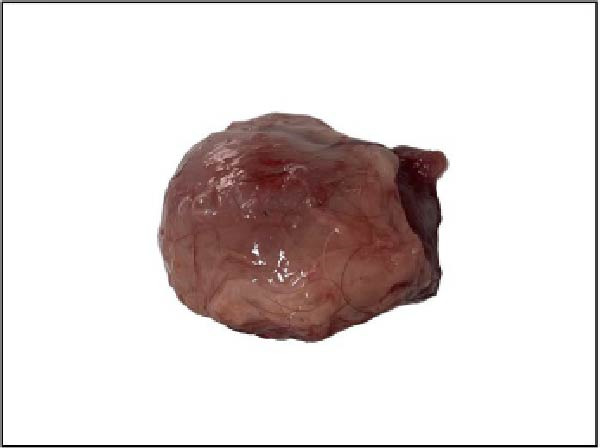
(B)

**Figure:**
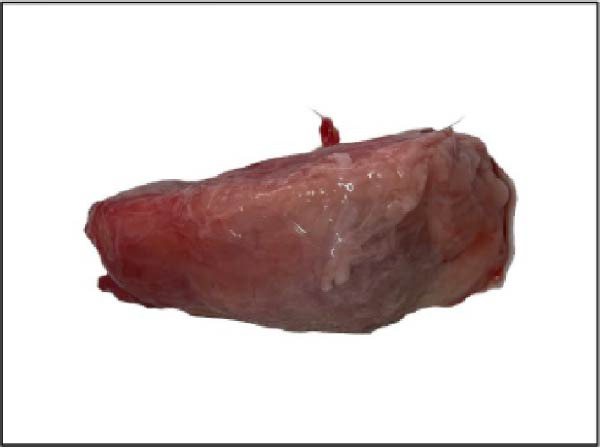
(C)

**Figure:**
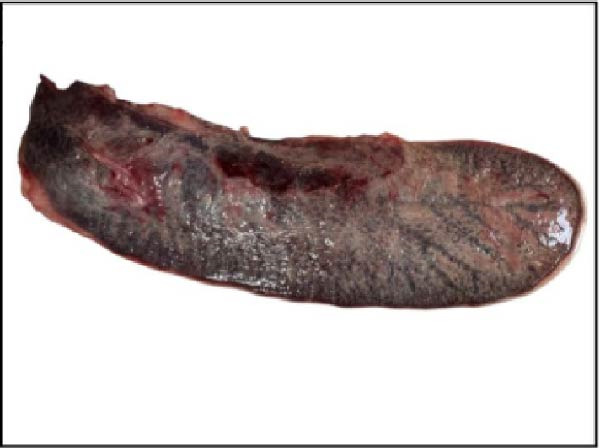
(D)

The nasal passages exhibited reddening and edema of the nostrils accompanied by mucus accumulation, indicating inflammatory changes. The tracheal mucosa was markedly congested with focal areas of necrosis (Figure 2A). In the lungs, bilateral lesions were observed, with ~60% of the apical lobes showing severe hepatizationconsistent with advanced pneumonia, which was evident in all six calves (Figure 2B). The myocardium appeared pale with multiple necrotic foci suggestive of infarction (Figure 2C). Gross examination of the kidneys revealed generalized congestion along with focal necrotic areas (Figure 2D). The myocardial and kidney lesions were found in Cases 2, 4, and 6 (Table 1).

Figure 2Postmortem findings in calves affected by lumpy skin disease (LSD) virus. (A) The tracheal mucosa exhibits large, round areas of focal necrosis surrounded by hemorrhagic zones. (B) Hepatization is observed in both lung lobes. (C) Multiple focal myocardial necrosis (infarct) in the heart (black circles). (D) Multiple focal necrosis in the kidney (black circle).

**Figure:**
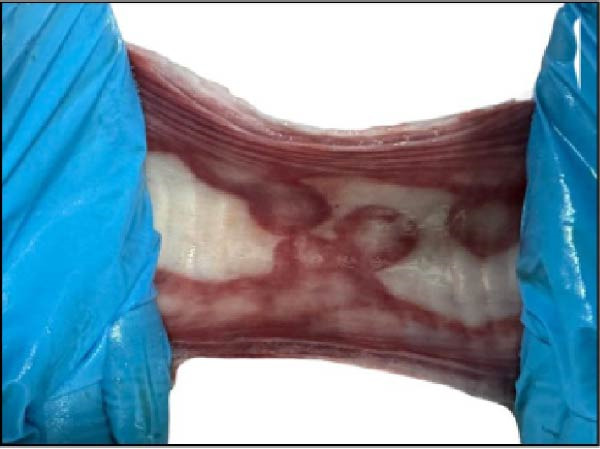
(A)

**Figure:**
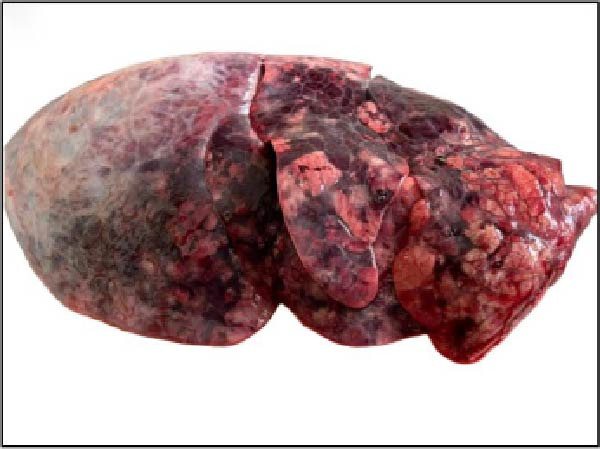
(B)

**Figure:**
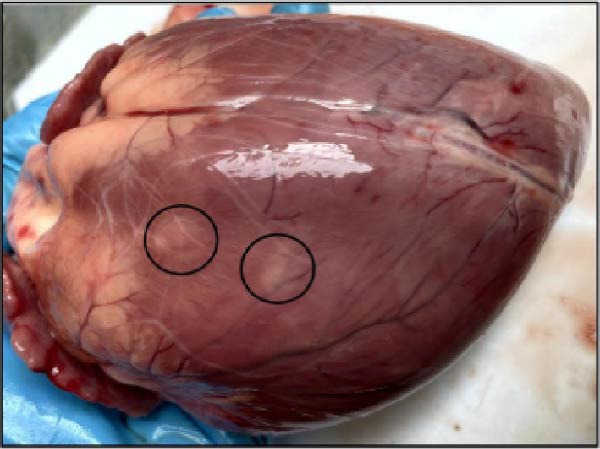
(C)

**Figure:**
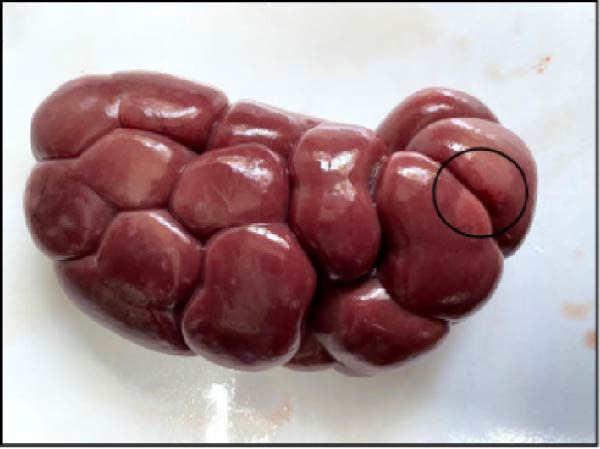
(D)

Further gross pathological examination of the gastrointestinal system revealed numerous nodules on the ruminal wall, visible from both the mucosal (3A) and serosal surfaces (Figure 3B). The lesions suggested chronic pathological processes and were observed in single deceased calf (Case 6; Table 1). Additionally, the liver was markedly enlarged with firm adhesions of the capsule and multifocal areas of necrosis distributed throughout the parenchyma (Figure 3C). The gall bladder appeared congested and showed multifocal necrotic lesions (3D). The spleen was atrophied, suggesting chronic systemic illness or immune suppression. Generalized lymph node enlargement was noted for all six cases (Table 1), with the brachial lymph node being prominently enlarged, consistent with a systemic inflammatory or infectious process. No gross pathological changes were observed in the brain.

Figure 3Postmortem findings in calves affected by lumpy skin disease (LSD) virus. (A, B) Numerous nodules observed on the ruminal wall: (A) mucosal surface and (B) serosal (outer) surface. (C) Liver exhibiting enlargement with capsular adhesion (arrowhead) with multiple necrotic foci (black circle). (D) Gallbladder exhibiting areas of focal necrosis (black circle).

**Figure:**
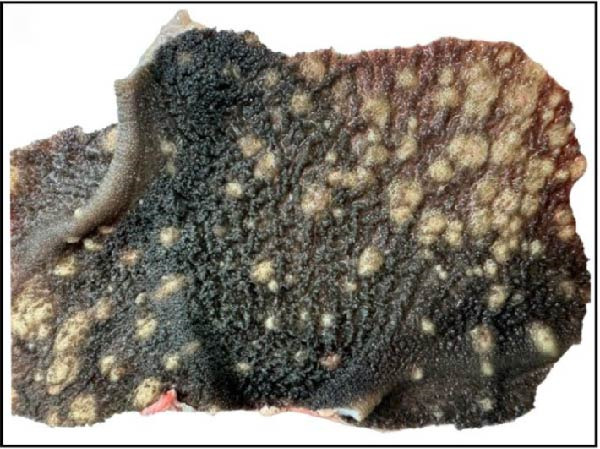
(A)

**Figure:**
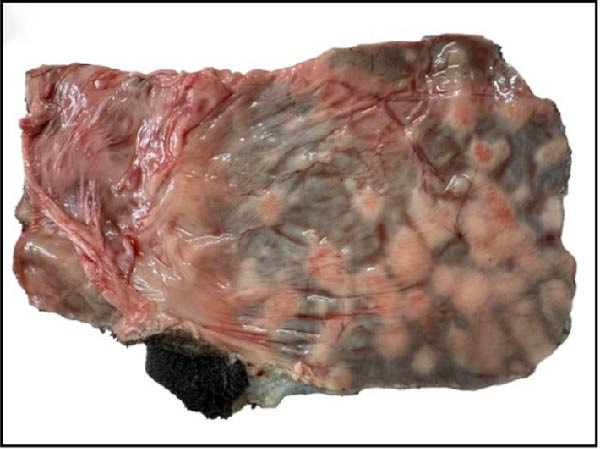
(B)

**Figure:**
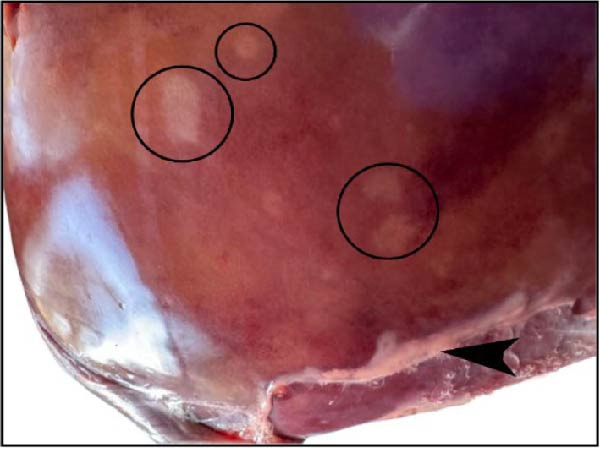
(C)

**Figure:**
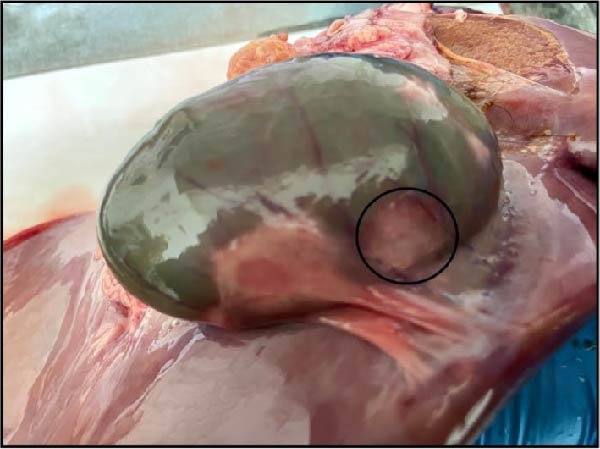
(D)

### 3.3. Histopathology

Histopathological examination of tissues from animals infected with LSDV revealed significant lesions across multiple organs. Necrotizing rhinitis was observed in the renal passage characterized by extensive perivascular inflammation, predominantly composed of lymphocytes (Figure 4A). Lung tissue showed severe chronic broncho‐interstitial pneumonia, with marked peribronchitis, indicating prolonged inflammatory involvement (Figure 4B). The liver exhibited hepatitis with prominent centrilobular necrosis, suggestive of virus‐induced hepatocellular damage (Figure 4C). In the gall bladder, perivasculitis was observed along with necrosis in the surrounding tissues, reflecting vascular inflammation and associated tissue injury (Figure 4D). These findings highlight the systemic nature of the involvement of the LSDV infection.

Figure 4Histopathological findings in the nasal passage, lung, liver, and gall bladder of calves infected by lumpy skin disease (LSD) virus. (A) Nasal passage exhibiting necrotizing rhinitis (black circle). (B) Lung tissue showing severe chronic broncho‐interstitial pneumonia. (C) Liver displaying hepatitis with centrilobular necrosis. (D) Gallbladder showing perivasculitis with surrounding tissue. The scale bar indicates magnification.

**Figure:**
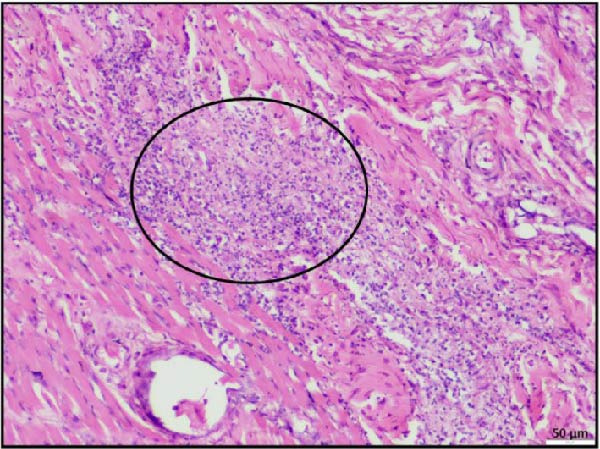
(A)

**Figure:**
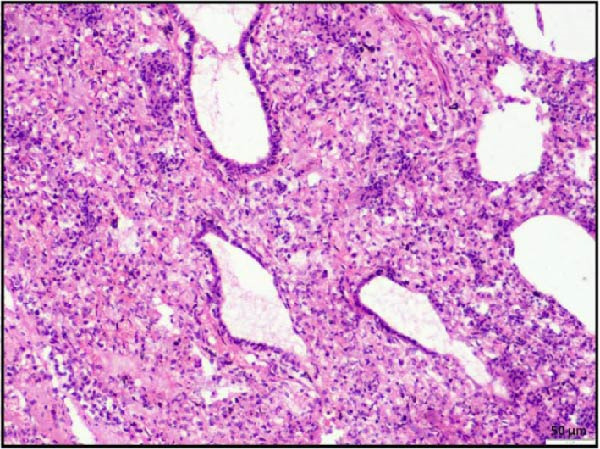
(B)

**Figure:**
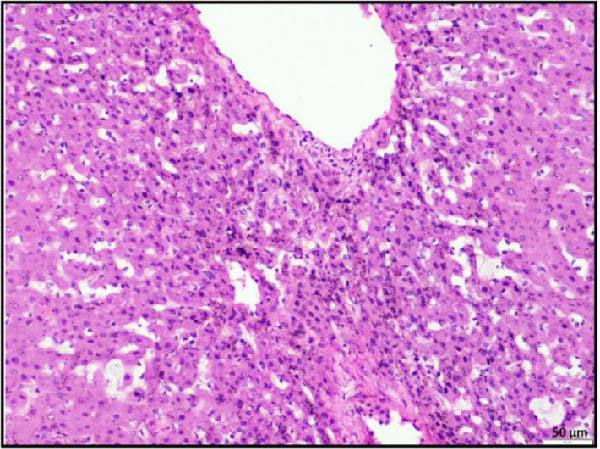
(C)

**Figure:**
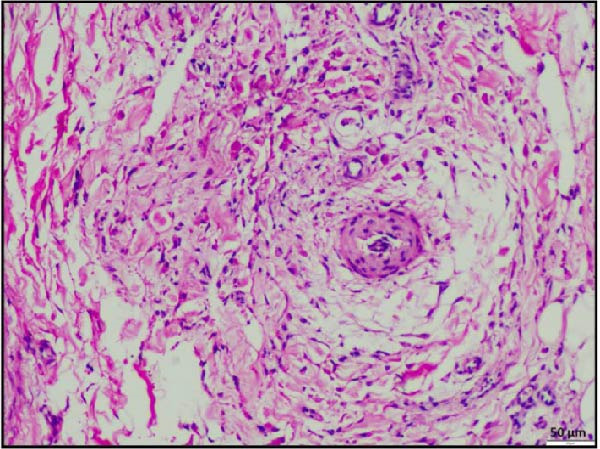
(D)

Histopathological examination of the rumen and heart in calves infected with the LSDV revealed distinct pathological changes. Well‐demarcated nodular aggregates were identified within the ruminal wall and, on histological examination, exhibited marked proliferation of dense connective tissue fibers accompanied by mild, discrete infiltration of lymphocytes and macrophage, consistent with vasculitis (Figure 5A,B). These features are consistent with a chronic inflammatory response. Further staining with Masson’s trichrome revealed mostly dense collagen fiber accumulation (Figure 5B) within these nodules, highlighting the elastic nature of the nodule, which may contribute to the restoration of normal architecture once the underlying cause is resolved. In the heart, the ventricular myocardium displayed severe localized necrosis with hemorrhages (infarct), with adjacent pale regions containing necrotic myocardial fibers, suggestive of localized ischemic damage (Figure 5C). These necrotic areas were clearly delineated and characterized by degeneration of muscle fibers (Figure 5D), confirming ischemic myocardial injury (infarct).

Figure 5Histopathological findings in the rumen and heart of calves infected by the lumpy skin disease (LSD) virus. (A) Ruminal nodule in the rumen wall exhibiting extensive proliferation of connective tissue with mild lymphocytic infiltration. (B) Masion’s trichrome stain highlighting proliferating collagen fibers (stained blue) in the nodule. (C) Ventricular myocardium displaying severe hemorrhages surrounded by a pale area with necrotic muscle fibers (arrowhead). (D) Ventricular muscle exhibiting a pale area with necrotic muscle fibers (highlighted box). The scale bar indicates magnification.

**Figure:**
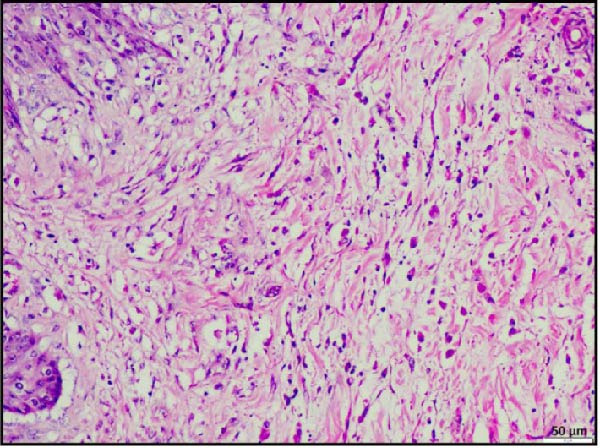
(A)

**Figure:**
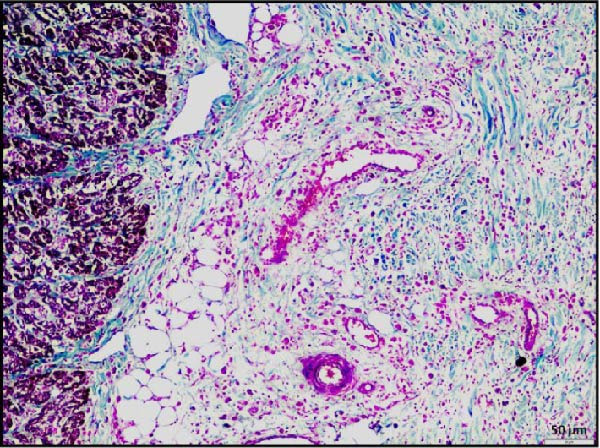
(B)

**Figure:**
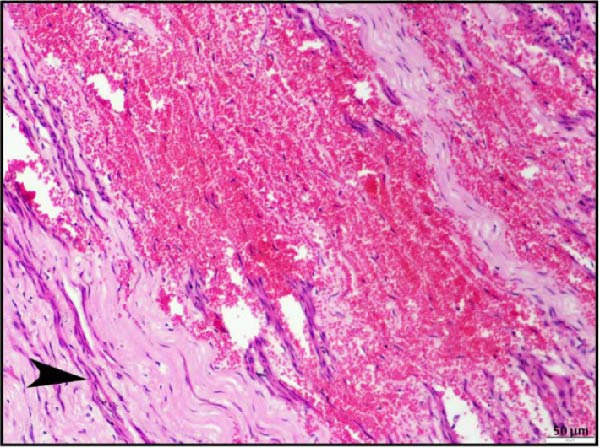
(C)

**Figure:**
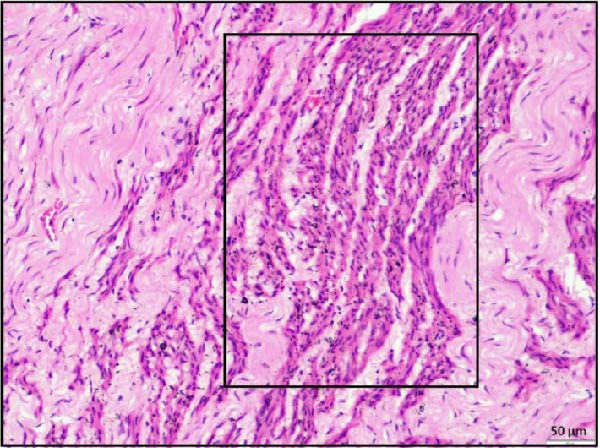
(D)

In the kidney, interstitial nephritis was observed, accompanied by vasculitis, as evidenced by inflammatory cell infiltration around blood vessels (Figure 6A). Additionally, focal areas of renal parenchymal necrosis were noted, suggesting localized tissue damage (Figure 6B). These findings demonstrate that LSDV infection can lead to significant necrotic changes in both gastrointestinal and cardiac tissues, indicating a broader systemic involvement than previously recognized. Histopathological analysis of the spleen and lymph nodes from animals infected with LSDV revealed significant tissue alterations indicative of systemic involvement. The spleen exhibited marked lymphoid depletion along with prominent trabecular thickening, indicative of a potentially compromised immune response and chronic inflammation (Figure 6C). Examination of the lymph nodes revealed extensive lymphoid necrosis, reflecting severe immunosuppression or direct viral CPEs (Figure 6D).

Figure 6Histopathological findings in the kidney, spleen, and lymph node of calves infected with lumpy skin disease (LSD) virus. (A) Kidney showing interstitial nephritis with vasculitis (black circle). (B) Kidney displaying focal necrosis (black circle). (C) Spleen exhibiting marked lymphoid depletion with prominent trabecular thickening. (D) Lymph node displaying extensive lymphoid necrosis. The scale bar indicates magnification.

**Figure:**
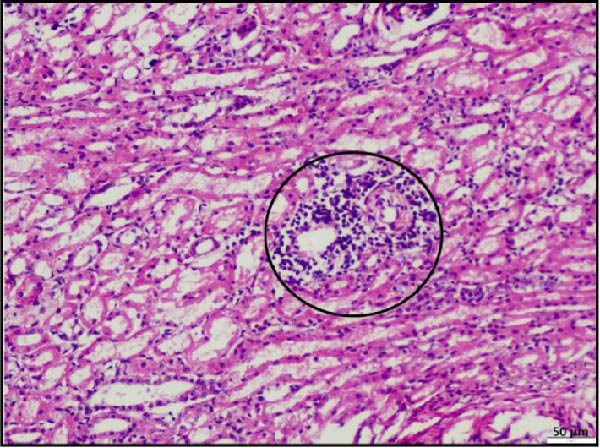
(A)

**Figure:**
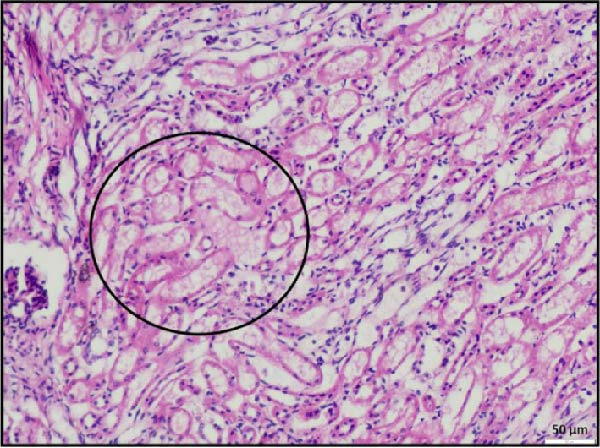
(B)

**Figure:**
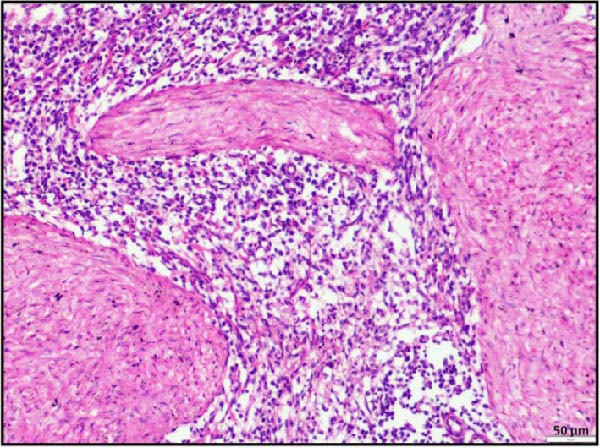
(C)

**Figure:**
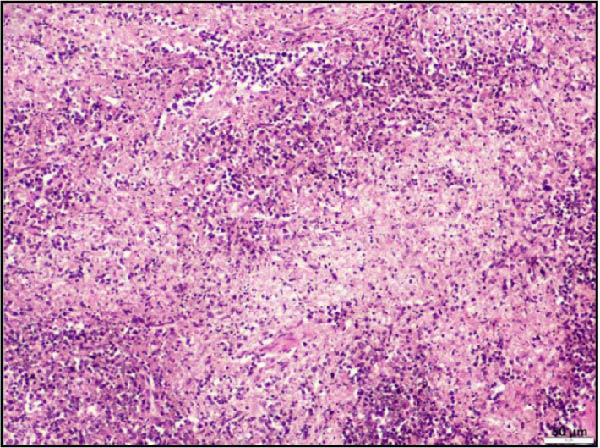
(D)

In summary, all these findings collectively underscore the multisystemic and immunopathological nature of LSDV infection, extending beyond cutaneous lesions.

### 3.4. Viral Load in Organs

**Figure 7:**
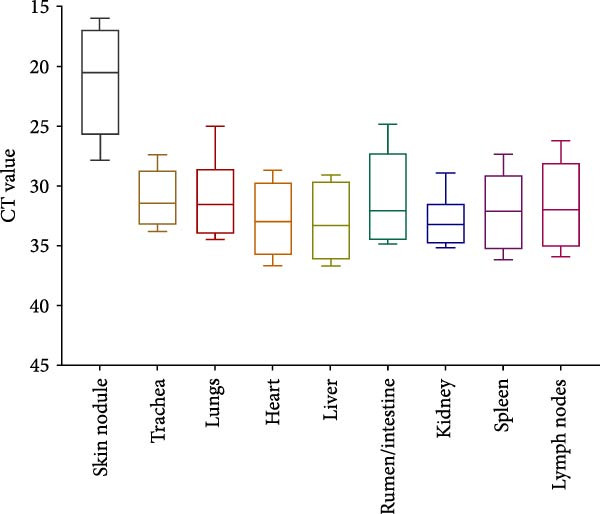
The distribution of LSD viruses in various organs of naturally infected deceased calves. The plot indicates the range of viral loads in skin and internal organs. The figure illustrates the systemic spread of LSD viruses across key visceral organs.

Skin nodules, trachea, lungs, hearts, liver, rumen, intestines, kidneys, spleens, and lymph node tissues were assessed for viral load using the pan‐Capripoxvirus qPCR approach that has been previously described [22]. The skin lesions had the highest LSD viral DNA load, with values ranging from 15.9 to 27.8. Ct values for other tissues are as follows: trachea 27.4–33.8, lungs 25–34.4, heart 28.7–36.6, liver 29–36.7, rumen and intestine 24.8–34.8, kidney 28.9–35.1, spleen 27.4–36.1, and lymph nodes 26.3–36 (Figure 7).

### 3.5. Virus Isolation

In the MDBK cell line, CPEs began to appear at 5 days postinfection (dpi) with LSDV (Figure 8A), while no morphological changes were observed in the negative control. By 7 dpi, the LSDV‐infected cells exhibited complete CPE (Figure 8C), whereas the control cells remained unaffected, showing no evidence of cytopathology (Figure 8B,D).

Figure 8Cytopathic effects (CPEs) in the MDBK cell line following LSDV infection compared to the negative control. (A) Initial CPE observed at 5 days postinfection (dpi), indicated by cellular rounding and detachment. (B) Negative control at 5 dpi shows an intact and confluent cell monolayer. (C) Extensive CPE observed at 7 dpi, with widespread cell degeneration and detachment. (D) Negative control at 7 dpi, maintaining normal cell morphology, and confluency.

**Figure:**
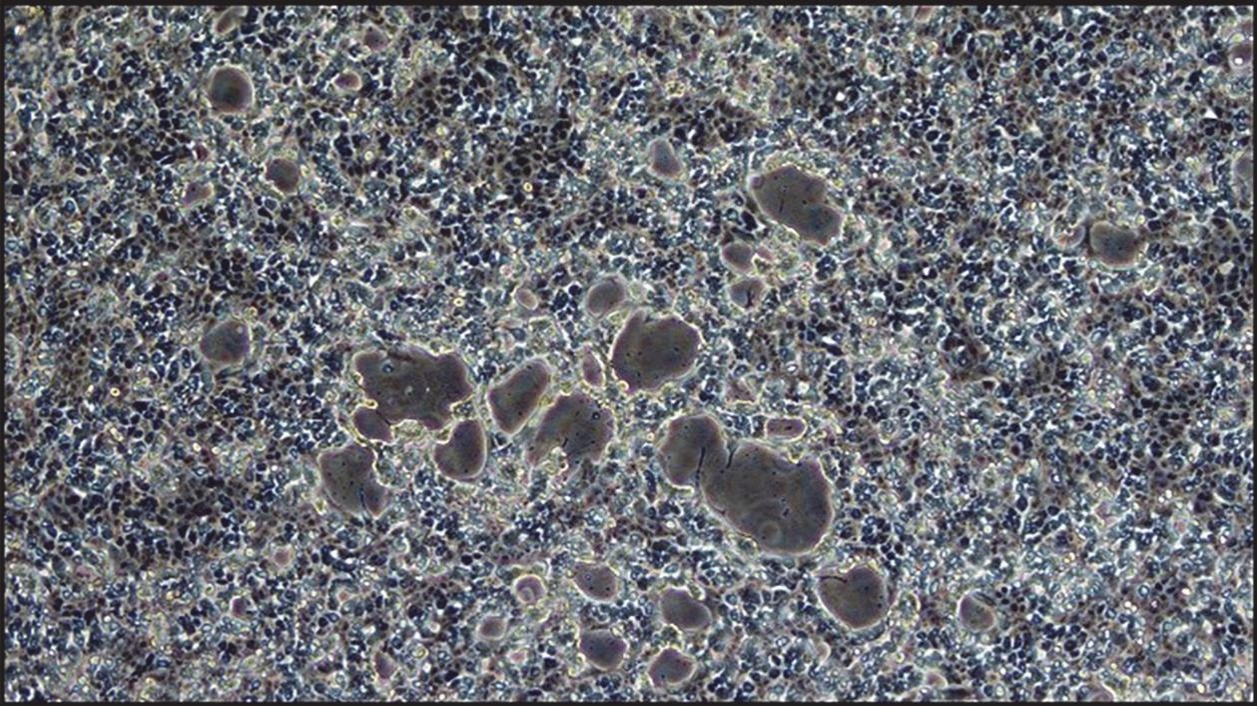
(A)

**Figure:**
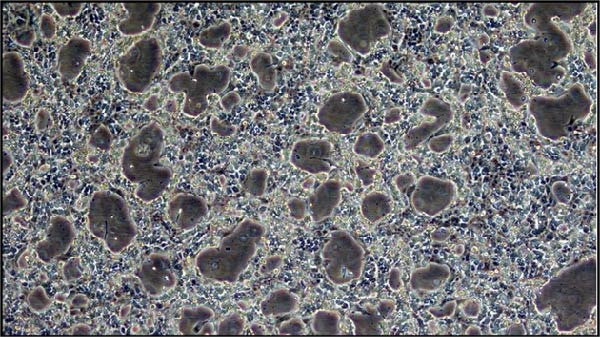
(B)

**Figure:**
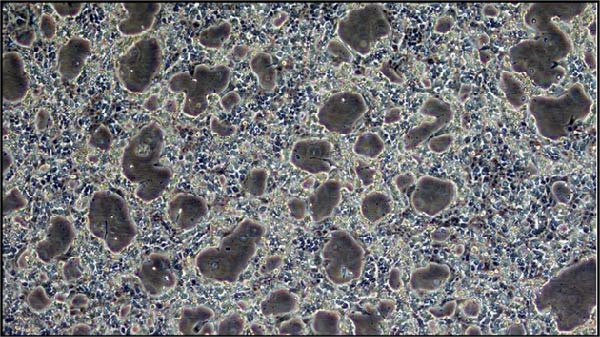
(C)

**Figure:**
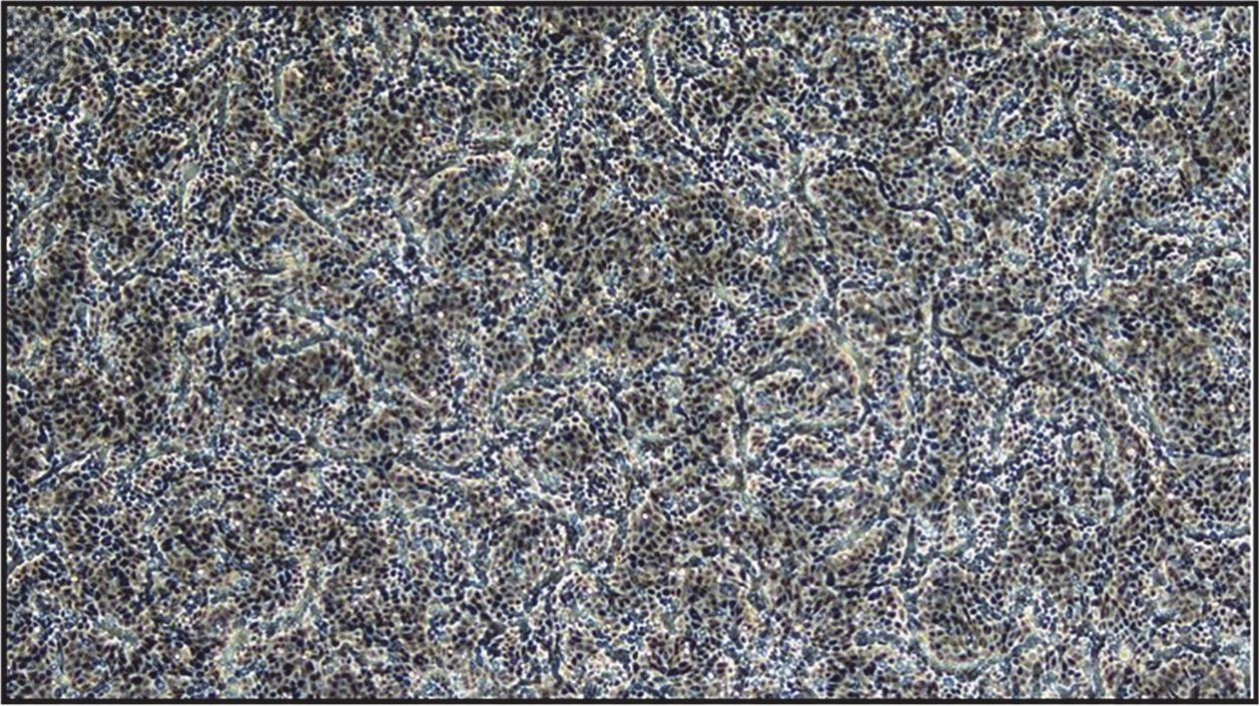
(D)

### 3.6. Phylogenic Analysis

**Figure 9:**
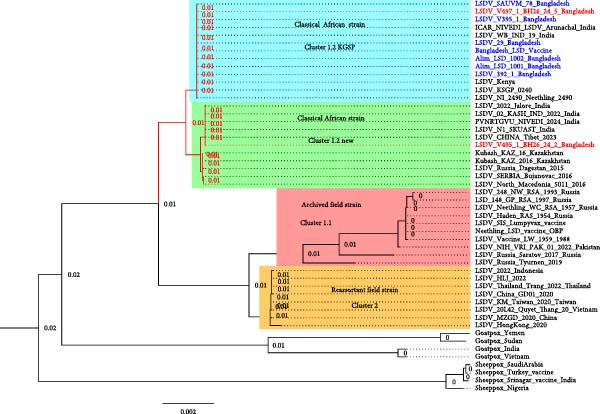
Phylogenetic analysis of circulating LSDV strains in Bangladesh. The maximum likelihood tree was constructed using whole genome sequences and illustrates the genetic relationship of Bangladeshi isolates with representative strains from different LSDV clusters, as well as with sheep pox virus (SPPV) and goat pox virus (GTPV) reference sequences.

After successful isolation, two LSDV strains were selected for whole‐genome sequencing based on their origin from two different outbreak locations. The sequences were completed by a commercial provider using standard procedures. The raw data were then used for mapping. The sequencing depth was comprehensive and good. An average sequence depth of 97.7 was determined for V495.1 and an average sequence depth of 146.1 for V497.1. To determine the genetic relationships of circulating strains, LSDV sequences available in GenBank were analyzed. Phylogenetic analysis revealed that cluster 1.1 comprises archived field isolates as well as commercial live vaccine strains, whereas cluster 1.2 represents the classical African strains and is further subdivided into two groups: the classical Kenyan sheep and goat pox (KSGP) lineage and a subgroup here referred to as “cluster 1.2 new,” which includes field isolates from Africa, the Middle East, Turkey, Serbia, Southern Europe, and Russia (Figure 9).

Notably, the two newly characterized Bangladeshi strains (v495‐2 and v497‐5; red taxa) were also grouped within cluster 1.2 the classical African strain group. Among them, strain v495‐2 clustered with “cluster 1.2 new,” formed a distinct branch closely related to strains from India, Macedonia, Serbia, and Russia, while strain v497‐5, together with previously characterized Bangladeshi strains (blue taxa), branched within the older classical African strain cluster 1.2 (KSGP lineage). The separation within cluster 1.2 was further supported by the identification of 250 single‐nucleotide polymorphisms (SNPs).

In contrast, cluster 2 consists of naturally occurring recombinant LSDV strains that were reported in Russia and Kazakhstan between 2017 and 2020. Importantly, none of the Bangladeshi strains characterized to date have grouped within these recombinant clusters. The successfully obtained sequences have been deposited in the GenBank database under the Accession numbers PX453692 and PX453693.

## 4. Discussion

LSDV infection in calves caused extensive necrotic lesions in multiple visceral organs (heart, lungs, liver, kidneys, rumen, and gallbladder), characterized histologically by diffuse to nodular necrotizing inflammation, infarction, and severe myocardial degeneration. This study demonstrated marked lymphocytic infiltration within the walls and perivascular areas of several organs, including the kidney, heart, ruminal wall, and gall bladder. Notably, a similar pattern of lesions was previously observed in the skin, as reported previously [2]. Such dominance of vascular‐associated lesions underscores vasculitis as a key driver of disease pathogenesis. The present study provides a comprehensive investigation of the clinical presentation, pathological changes, viral isolation, tissue tropism, and molecular characterization of naturally occurring LSDV, particularly in fatal calf cases from several regions of Bangladesh.

LSD is a highly contagious, eruptive, and economically significant disease affecting cattle. It was first reported in Bangladesh in July 2019 and rapidly spread to several districts [9]. Since then, the virus has continued to circulate widely within the national cattle population [2]. The clinical signs and skin lesions documented in this study (skin nodules, lymphadenopathy, and edema) align with those reported in previous studies from various countries [2, 13, 24–27]. Additional clinical observations, including hypersalivation and nasal and ocular discharges, align with documented clinical manifestations [28, 29].

Gastrointestinal lesions, including nodules on both the mucosal and serosal surfaces of the ruminal wall, are a relatively recent finding, although nodular lesions have been described in the alimentary tract of experimentally infected animals [30]. The gall bladder also contained multifocal necrotic lesions, which, to our knowledge, are rarely observed in LSDV pathological studies. Due to viremia, viruses become distributed across various organs, showing affinity to endothelial and smooth blood vessel cells, as previously described in the histopathology of skin lumps [2]. This vascular involvement limits blood circulation in the adjacent tissues, leading to hypoxia driven cell death, inflammation, and subsequent nodular proliferation of collagen fibers to repair the necrosed areas. Notably, the observation of multifocal necrosis resembling infarction and associated tissue damage in both heart and kidneys is remarkable, as such lesions represent severe tissue pathology and have been rarely documented in cases of LSDV infection. These findings broaden the recognized tissue tropism of LSDV and hold important implications for understanding viral persistence and systemic pathogenicity.

Histopathological findings further confirmed the systemic nature of LSDV infection. In addition to the characteristic skin lesions previously reported [2, 31], the present report describes widespread histopathological changes in multiple organ systems, such as the respiratory, gastrointestinal, cardiac, renal, and lymphoid systems, which attest to the extensive pathological impact of LSDV. Infarction in vital organs such as the heart and kidneys can critically impair organ function and is therefore potentially fatal in any species of animals. Necrotizing rhinitis, interstitial pneumonia, and bronchitis in the respiratory tract were prominent lesions that correlate well with respiratory distress (dyspnea) observed in affected calves. The liver exhibited hepatitis characterized by centrilobular necrosis, diffuse and hepatocellular degeneration correlated with gross findings, and reflecting systemic clinical involvement. Lesions resembling cystitis were observed in the gallbladder, while the ruminal wall contained nodular proliferation composed of connective tissue with mild inflammatory infiltration. To clarify the underlying nature of these nodules, whether they resulted from granulomatous proliferation, intradermal accumulation of collagen and elastic fibers, or submucosal connective tissue expansion in the rumen, Masson’s trichrome stain was employed. Masson’s trichrome staining of rumen nodules revealed extensive deposition of collagen fiber, indicative of chronic inflammation and tissue remodeling associated with persistent viral infection. The concurrent occurrence of these changes, together with vasculitis, highlights hematogenous dissemination of the virus to different systems. PCR confirmation of viral genomes in these tissues establishes a direct causal link between LSDV and the lesions. Notably, such widespread digestive tract pathology has not been reported in earlier studies. These findings, therefore, extend the recognized tissue tropism of LSDV and suggest a more complex systemic pathogenesis than previously understood. Involvement of the rumen and gallbladder raises new questions about viral persistence, chronicity, and potential shedding pathways, with important implications for transmission dynamics and long‐term carrier status.

Most notably, myocardial histology revealed hemorrhagic infarction accompanied by degeneration of surrounding cardiac muscle fibers. Such lesions are uncommon in natural LSDV outbreaks, indicating that localized ischemia, likely secondary to vasculopathy, may be the underlying cause. Renal histopathology revealed interstitial nephritis, vasculitis, and focal necrosis, supporting the hypothesis further that vasculitis induced by LSDV can lead to ischemic injury in other organs. Although the same findings were reported in an experimental study [32], the study failed to describe lesions in naturally occurring LSDV.

The highest viral load was detected in skin nodules, consistent with previous reports [1, 2, 11, 22, 31, 33]. Notably, substantial concentrations of LSDV DNA were also detected in visceral organs, including heart, liver, and kidneys. Detection of substantial viral DNA in visceral organs indicates systemic dissemination through viremia. Such viremia allows the virus to reach multiple organ systems, contributing to widespread tissue involvement, increased severity of clinical signs, and ultimately the fatal outcomes observed in these calves.

The present study also provides important insights into the molecular epidemiology of LSDV circulating in Bangladesh. Whole‐genome sequencing and phylogenetic analysis revealed a transition from cluster 1.1 to cluster 1.2, suggesting mutation‐driven evolution. This genetic transition is of particular concern, as cluster 1.2 (classical African‐like strains) has been associated with higher virulence and increased calf mortality [18]. The accumulation of a considerable number of SNPs within this cluster further indicates ongoing viral diversification, which may contribute to enhanced pathogenicity and complicated disease management.

Earlier studies in Bangladesh reported the circulation of older African strains [2, 34]. Our findings, however, demonstrate the continued presence of cluster 1.2 viruses, with some strains forming distinct branches within this lineage. Interestingly, the two newly characterized strains (v495‐2 and v497‐5) exhibited a notable genetic divergence, supported by more than 250 SNP differences. Such intra‐cluster heterogeneity highlights the ongoing viral evolution within Bangladesh and suggests multiple introductions or independent evolutionary trajectories or local evolutionary adaptation under selective pressures such as host immunity, vaccination, or ecological factors. Such genetic variation is of epidemiological significance, as it may reflect viral evolution and adaptation, potentially driving differences in virulence and transmission dynamics. Our observation of multisystemic pathology caused by LSDV strains, extending beyond the predominantly cutaneous lesions reported in earlier outbreaks, provides further evidence of this evolutionary shift in pathogenic potential. These findings also raise important concerns regarding vaccine efficacy and control strategies, as viral adaptation may influence immune escape, alter clinical manifestations, and complicate surveillance and eradication efforts.

Importantly, none of the Bangladeshi strains analyzed in this study clustered with the recombinant lineages (clusters 2.1–2.6) that have been reported from Russia and Kazakhstan in recent years. Recombinant LSDV strains have raised concerns globally due to their altered biological properties and reduced vaccine protection [35]. Mutations, particularly in immunogenic regions of the LSDV genome such as the GPCR and RPO30 genes, could alter antigenic properties and potentially affect vaccine‐induced protection. Although our findings do not directly confirm vaccine escape, the observed genetic variations highlight the need for continuous genomic surveillance to monitor possible impacts on vaccine effectiveness.

Despite the absence of recombinant lineages, the observed genetic diversification within cluster 1.2 highlights the importance of ongoing genomic surveillance to monitor for the possible emergence or introduction of recombinant strains in near future. A few limitations of this study need to be highlighted. The sample size was small and was limited to fatal cases submitted for postmortem examination, and therefore, the sample may overrepresent the severity of the disease. Second, although we quantified viral DNA loads using qPCR, we did not perform virus titration or immunohistochemistry to ascertain viral antigen localization within tissues. Therefore, future studies should include a larger sample size, quantitative virus titration, and immunohistochemistry to better correlate viral load with lesion severity and tissue tropism.

All six calves included in the study were clinically and pathologically confirmed cases of LSD. PCR testing ruled out coinfections with other major bovine viral pathogens, including foot‐and‐mouth disease (FMD) virus. Additionally, no pre‐existing health issues were reported by the farm owners. Therefore, the observed lesions are considered primarily associated with LSDV infection. An attenuated goat pox virus (GTPV) vaccine, locally produced by the Livestock Research Institute (LRI), Bangladesh, is currently used by the government for LSD control; however, its coverage remains limited, leaving many smallholders unvaccinated. In addition, a few commercial vaccines Lumpyvax (MSD Animal Health) and BOVIVAX LSD (ACI, Bangladesh) have recently been introduced to the Bangladeshi market, though their use is largely confined to large‐scale farms. Most of these vaccines are recommended for administration at 6 months of age or older; therefore, younger calves remain unprotected unless sufficient maternal antibodies are present. In our study, the affected calves were unvaccinated, and maternal antibody protection was likely absent. Therefore, widespread systemic clinical and pathological alterations were evident in the affected calves.

Taken together, our findings underscore the complex pathogenesis of LSDV and its potential to cause multisystemic disease with widespread necrotic, vascular, and fibrotic lesions extending beyond the skin. Vasculitis emerges as a key driver of pathology, while the involvement of vital visceral organs such as the heart, kidneys, and liver likely contributes to fatal outcomes in calves. Moreover, the circulating LSDV strains in Bangladesh are undergoing genetic diversification within the classical cluster 1.2 lineage. In conclusion, LSDV in Bangladesh is evolving within cluster 1.2 and poses a transboundary threat through its vasculopathy‐driven multisystemic pathology, leading to fatal visceral involvement in calves.

## Ethics Statement

The “Ethical Standard of Research Committee” (135/BAURES/ESRC/VET/23), Bangladesh Agricultural University, Mymensingh, reviewed and approved the procedures used, and the animals were treated with care.

## Conflicts of Interest

The authors declare no conflicts of interest.

## Author Contributions

**Israt Jerin, Md. Riabbel Hossain, and Emdadul Haque Chowdhury:** data curation, formal analysis, investigation, methodology, writing – original draft, writing – review and editing. **Shadia Tasnim:** data curation, investigation, methodology, writing – original draft, writing – review and editing. **Seikh Masudur Rahman:** data curation, formal analysis, investigation, writing – original draft. **Anja Globig and Bernd Hoffman:** formal analysis, investigation, methodology, writing – original draft, writing – review and editing. **Rokshana Parvin:** conceptualization, data curation, formal analysis, investigation, methodology, writing – original draft, writing – review and editing, funding acquisition, resources, supervision. Israt Jerin and Md. Riabbel Hossain contributed equally to this work.

## Funding

The funding for the study comes from the Department of Livestock Services, Ministry of Fisheries and Livestock, Bangladesh’s Livestock and Dairy Development Project (LDDP), subproject number RP‐C‐01‐03‐23, and technical support (sequencing) from Friedrich Loeffler Institute in Germany.

## Data Availability

All required data are available as figures and table in the main text.

## References

[1] Adamu K. , Abayneh T. , and Getachew B. , et al.Lumpy Skin Disease Virus Isolation, Experimental Infection, and Evaluation of Disease Development in a Calf, Scientific Reports. (2024) 14, no. 1, 10.1038/s41598-024-60994-8, 20460. 39227598 PMC11372140

[2] Parvin R. , Chowdhury E. H. , and Islam M. T. , et al.Clinical Epidemiology, Pathology, and Molecular Investigation of Lumpy Skin Disease Outbreaks in Bangladesh during 2020–2021 Indicate the Re-Emergence of an Old African Strain, Viruses. (2022) 14, no. 11, 10.3390/v14112529, 2529. 36423138 PMC9698944

[3] Tuppurainen E. S. M. , Babiuk S. , and Klement E. , Lumpy Skin Disease, 2018, Springer International Publishing, 10.1007/978-3-319-92411-3, 2-s2.0-85063363093.

[4] Di Giuseppe A. , Zenobio V. , Dall’Acqua F. , Di Sabatino D. , and Calistri P. , Lumpy Skin Disease, Veterinary Clinics of North America: Food Animal Practice. (2024) 40, no. 2, 261–276, 10.1016/j.cvfa.2024.01.002. 38811129

[5] Tasioudi K. E. , Antoniou S. E. , and Iliadou P. , et al.Emergence of Lumpy Skin Disease in Greece, 2015, Transboundary and Emerging Diseases. (2016) 63, no. 3, 260–265, 10.1111/tbed.12497, 2-s2.0-84961285120. 26991342

[6] Abutarbush S. M. , Ababneh M. M. , and Al Zoubi I. G. , et al.Lumpy Skin Disease in Jordan: Disease Emergence, Clinical Signs, Complications and Preliminary-Associated Economic Losses, Transboundary and Emerging Diseases. (2015) 62, no. 5, 549–554, 10.1111/tbed.12177, 2-s2.0-84940583410. 24148185

[7] Namazi F. and Khodakaram Tafti A. , Lumpy Skin Disease, an Emerging Transboundary Viral Disease: A Review, Veterinary Medicine and Science. (2021) 7, no. 3, 888–896, 10.1002/vms3.434. 33522708 PMC8136940

[8] Zeynalova S. , Asadov K. , Guliyev F. , Vatani M. , and Aliyev V. , Epizootology and Molecular Diagnosis of Lumpy Skin Disease among Livestock in Azerbaijan, Frontiers in Microbiology. (2016) 7, 10.3389/fmicb.2016.01022, 2-s2.0-84980337498, 1022. 27446057 PMC4926614

[9] Hasib F. M. Y. , Islam M. S. , and Das T. , et al.Lumpy Skin Disease Outbreak in Cattle Population of Chattogram, Bangladesh, Veterinary Medicine and Science. (2021) 7, no. 5, 1616–1624, 10.1002/vms3.524. 33993641 PMC8464269

[10] Chouhan C. S. , Parvin M. S. , and Ali M. Y. , et al.Epidemiology and Economic Impact of Lumpy Skin Disease of Cattle in Mymensingh and Gaibandha Districts of Bangladesh, Transboundary and Emerging Diseases. (2022) 69, no. 6, 3405–3418, 10.1111/tbed.14697. 36056232

[11] Giasuddin M. , Yousuf M. A. , Hasan M. , Rahman M. H. , Hassan M. Z. , and Ali M. Z. , Isolation and Molecular Identification of Lumpy Skin Disease (LSD) Virus From Infected Cattle in Bangladesh, Bangladesh Journal of Livestock Research. (2020) 26, no. 1-2, 15–20, 10.3329/bjlr.v26i1-2.49933.

[12] Wani A. A. , Clinical Management of Lumpy Skin Disease (LSD) in Cattle, International Journal of Current Microbiology and Applied Sciences. (2022) 11, no. 10, 196–206, 10.20546/ijcmas.2022.1110.024.

[13] Kumar N. , Sharma S. , and Tripathi B. N. , Pathogenicity and Virulence of Lumpy Skin Disease Virus: A Comprehensive Update, Virulence. (2025) 16, no. 1, 10.1080/21505594.2025.2495108, 2495108. 40265421 PMC12036493

[14] Mathewos M. , Dulo F. , Tanga Z. , and Sombo M. , Clinicopathological and Molecular Studies on Cattle Naturally Infected with Lumpy Skin Diseases in Selected Districts of Wolaita Zone, Southern Ethiopia, BMC Veterinary Research. (2022) 18, no. 1, 10.1186/s12917-022-03403-4, 297. 35922813 PMC9347132

[15] Bowden T. R. , Babiuk S. L. , Parkyn G. R. , Copps J. S. , and Boyle D. B. , Capripoxvirus Tissue Tropism and Shedding: A Quantitative Study in Experimentally Infected Sheep and Goats, Virology. (2008) 371, no. 2, 380–393, 10.1016/j.virol.2007.10.002, 2-s2.0-38849183418. 17988703 PMC9955785

[16] Fay P. C. , Wijesiriwardana N. , and Munyanduki H. , et al.The Immune Response to Lumpy Skin Disease Virus in Cattle Is Influenced by Inoculation Route, Frontiers in Immunology. (2022) 13, 10.3389/fimmu.2022.1051008, 1051008. 36518761 PMC9742517

[17] LaTourrette K. and Garcia-Ruiz H. , Determinants of Virus Variation, Evolution, and Host Adaptation, Pathogens. (2022) 11, no. 9, 10.3390/pathogens11091039, 1039. 36145471 PMC9501407

[18] Xie S. , Cui L. , and Liao Z. , et al.Genomic Analysis of Lumpy Skin Disease Virus Asian Variants and Evaluation of Its Cellular Tropism, npj Vaccines. (2024) 9, no. 1, 10.1038/s41541-024-00846-8. PMC10957905 38514651

[19] Tuppurainen E. , Dietze K. , and Wolff J. , et al.Review: Vaccines and Vaccination against Lumpy Skin Disease, Vaccines. (2021) 9, no. 10, 10.3390/vaccines9101136. PMC8539040 34696244

[20] Parvin R. , Al Mim S. , and Haque M. N. , et al.Serological Response to Lumpy Skin Disease in Recovered and Clinically Healthy Vaccinated and Unvaccinated Cattle of Bangladesh, Frontiers in Veterinary Science. (2025) 12, 10.3389/fvets.2025.1535600, 1535600. 40034563 PMC11873106

[21] Dietze K. , Moritz T. , and Alexandrov T. , et al.Suitability of Group-Level Oral Fluid Sampling in Ruminant Populations for Lumpy Skin Disease Virus Detection, Veterinary Microbiology. (2018) 221, 44–48, 10.1016/j.vetmic.2018.05.022, 2-s2.0-85047855237. 29981707

[22] Pervin S. , Ahamed M. , and Chouhan C. S. , et al.Isolation, Adaptation, and Characterization of Lumpy Skin Disease Virus from Cattle in Bangladesh, Journal of Advanced Veterinary and Animal Research. (2023) 10, no. 3, 563–569, 10.5455/javar.2023.j710. 37969804 PMC10636076

[23] Katoh K. and Standley D. M. , MAFFT Multiple Sequence Alignment Software Version 7: Improvements in Performance and Usability, Molecular Biology and Evolution. (2013) 30, no. 4, 772–780, 10.1093/molbev/mst010, 2-s2.0-84875619226. 23329690 PMC3603318

[24] R. Rouby S. , Shehata O. , S.Abdel-Moneim A. , H.Hussein K. , and Mahmoud M. M , Lumpy Skin Disease in Calves: The Association Between Clinical Signs and Biochemical Alterations, Advances in Animal and Veterinary Sciences. (2021) 9, no. 11, 1863–1868, 10.17582/journal.aavs/2021/9.11.1863.1868.

[25] Salib F. A. and Osman A. H. , Incidence of Lumpy Skin Disease among Egyptian Cattle in Giza Governorate, Egypt, Veterinary World. (2009) 4, no. 4, 162–167, 10.5455/vetworld.2011.162-167, 2-s2.0-84860456434.

[26] Ochwo S. , VanderWaal K. , and Munsey A. , et al.Seroprevalence and Risk Factors for Lumpy Skin Disease Virus Seropositivity in Cattle in Uganda, BMC Veterinary Research. (2019) 15, no. 1, 10.1186/s12917-019-1983-9, 2-s2.0-85069037172, 236. 31286926 PMC6615106

[27] Datten B. , Chaudhary A. A. , and Sharma S. , et al.An Extensive Examination of the Warning Signs, Symptoms, Diagnosis, Available Therapies, and Prognosis for Lumpy Skin Disease, Viruses. (2023) 15, no. 3, 10.3390/v15030604. PMC10058328 36992313

[28] Ratyotha K. , Prakobwong S. , and Piratae S. , Lumpy Skin Disease: A Newly Emerging Disease in Southeast Asia, Veterinary World. (2022) 2764–2771, 10.14202/vetworld.2022.2764-2771. 36718323 PMC9880836

[29] DLS , Guidelines for Clinical Management of Lumpy Skin Disease in Bangladesh Guidelines for Clinical Management of Lumpy Skin Disease in Bangladesh, 2021, Department of Livestock Services.

[30] Babiuk S. , Bowden T. R. , Boyle D. B. , Wallace D. B. , and Kitching R. P. , Capripoxviruses: An Emerging Worldwide Threat to Sheep, Goats and Cattle, Transboundary and Emerging Diseases. (2008) 55, no. 7, 263–272, 10.1111/j.1865-1682.2008.01043.x, 2-s2.0-49049110254. 18774991

[31] Amin D. M. , Shehab G. , and Emran R. , et al.Diagnosis of Naturally Occurring Lumpy Skin Disease Virus Infection in Cattle Using Virological, Molecular, and Immunohistopathological Assays, Veterinary World. (2021) 2230–2237, 10.14202/vetworld.2021.2230-2237. 34566343 PMC8448636

[32] Kutumbetov L. , Ragatova A. , and Azanbekova M. , et al.Investigation of the Pathogenesis of Lumpy Skin Disease Virus in Indigenous Cattle in Kazakhstan, Pathogens. (2025) 14, no. 6, 10.3390/pathogens14060577. PMC12196440 40559586

[33] Ochwo S. , VanderWaal K. , and Ndekezi C. , et al.Molecular Detection and Phylogenetic Analysis of Lumpy Skin Disease Virus from Outbreaks in Uganda 2017–2018, BMC Veterinary Research. (2020) 16, no. 1, 10.1186/s12917-020-02288-5, 66. 32085763 PMC7035724

[34] Badhy S. C. , Chowdhury M. G. A. , and Settypalli T. B. K. , et al.Molecular Characterization of Lumpy Skin Disease Virus (LSDV) Emerged in Bangladesh Reveals Unique Genetic Features Compared to Contemporary Field Strains, BMC Veterinary Research. (2021) 17, no. 1, 10.1186/s12917-021-02751-x, 61. 33514360 PMC7844896

[35] Sprygin A. , Pestova Y. , and Bjadovskaya O. , et al.Evidence of Recombination of Vaccine Strains of Lumpy Skin Disease Virus with Field Strains, Causing Disease, PLoS ONE. (2020) 15, no. 5, 10.1371/journal.pone.0232584. PMC7219772 32401805

